# Metabolic shift precedes the resolution of inflammation in a cohort of patients undergoing bariatric and metabolic surgery

**DOI:** 10.1038/s41598-021-91393-y

**Published:** 2021-06-09

**Authors:** Jose Romeo Villarreal-Calderon, Ricardo Cuellar-Tamez, Elena C. Castillo, Eder Luna-Ceron, Gerardo García-Rivas, Leticia Elizondo-Montemayor

**Affiliations:** 1 Tecnologico de Monterrey, Escuela de Medicina y Ciencias de la Salud, 64710 Monterrey, Mexico; 2 Tecnologico de Monterrey, Centro de Investigación en Obesidad y Nutrición Clínica, 64710 Monterrey, Mexico; 3 Tecnologico de Monterrey, Centro de Investigación Biomédica, Hospital Zambrano Hellion, TecSalud, 66278 San Pedro Garza García, Mexico; 4 Tecnologico de Monterrey. Cardiovascular Medicine and Metabolomics Research Group, Hospital Zambrano Hellion, TecSalud, 66278 San Pedro Garza García, Mexico

**Keywords:** Endocrinology, Molecular medicine, Pathogenesis

## Abstract

Bariatric and metabolic surgery has shown to promote weight loss and reduce systemic inflammation. However, the sequence and timing of events regarding metabolic improvement and inflammation resolution has been rarely explored. Furthermore, data on inflammatory markers of Th17 and Th1 cell responses after bariatric surgery is scarce. We conducted a prospective study in subjects with obesity that underwent bariatric and metabolic surgery, with follow-ups at 3 and 6 months. Anthropometric and metabolic markers such as insulin levels, HOMA-IR, and lipid parameters declined significantly 3 months after surgery; while hs-CRP, TNF-α, IL-1β, IL-6, and IL-8 serum concentrations decreased 6 months after the procedure. Concentrations of Th1 signature and driver cytokines, particularly IFN-γ, IL-12, and IL-18, and of Th17 driver IL-23 also decreased significantly after 6 months. Significant positive correlations between triglyceride levels and hs-CRP, IL-1β, and IFN-γ concentrations, and between Apo B and IFN-γ levels were observed 6 months after bariatric and metabolic surgery. In addition, BMI was associated with hs-CRP and TNF-α concentrations. Fat mass correlated with hs-CRP, TNF-α, and IL-12. Analysis of the temporality of metabolic and inflammatory events suggests that improvement in the metabolic status occurs before resolution of systemic inflammation and may be a requisite for the later event.

## Introduction

The worldwide prevalence of obesity has increased to the extent of becoming a public health concern and was estimated to be of 10.8% in men and 14.9% in women in 2014^1^. It has recently been reported to reach 39.6% in the US population^2^. Besides the well-studied cardiometabolic diseases such as type 2 diabetes mellitus (T2DM), dyslipidemias, and hypertension ^3^, obesity has also been characterized by chronic low-grade systemic inflammation^4^. The mechanisms driving the chronic low-grade inflammation in obesity are not completely understood. Novel insights indicate that metabolic markers, rather than classic immunologic activators, promote a pro-inflammatory shift in macrophages and lymphocytes^5,6^. On the other hand, obesity-associated inflammation induces insulin resistance^7^. Therefore, the effects of inflammation and metabolic changes on each other might be bidirectional.

Lifestyle modification through diet and physical activity is usually the first approach to treat obesity. However, the reduction in weight loss is generally mild and weight regain is common. In a large longitudinal study, including 5145 subjects with obesity and diabetes, only 50% of the patients were shown to maintain a weight loss of 5% after 1 year^8^. On the other hand, bariatric and metabolic surgery (BMS) has been proven to be an effective treatment for patients with a body mass index (BMI) above 40 kg/m^2^ or a BMI above 35 kg/m^2^ in the presence of comorbidities and failed previous conservative attempts to lose weight^9^. Also, BMS has been shown to be not only a viable therapy to accomplish supported weight reduction but also to promote metabolic improvement^10^. In this regard, BMS-induced weight loss has been associated with both remission of comorbidities and reduction in systemic inflammation^11^. High-sensitivity C-reactive protein (hs-CRP) levels were demonstrated to decrease significantly 3 months after surgery in subjects with obesity undergoing Roux-Y-gastroplasty (RYGB)^12^. Its levels were also found to decline one month after surgery, while IL-6 concentration was shown to diminish 6 months after the procedure in patients with obesity submitted to sleeve gastrectomy (SG)^13^. Similar findings were described in a recent meta-analysis that pooled results from more than 100 studies^14^. Circulatory levels of IL-1β, a key proinflammatory component of the inflammasome involved in the pathophysiology of insulin resistance, were also reported to decrease after weight loss surgery^15^.

BMS has been shown to lead to significant improvements in insulin resistance^16^ and lipid metabolism^17^, as well as to resolve the low-grade systemic inflammation associated with obesity^12^. However, the sequence of both events is not well characterized. In this sense, a direct comparison of the temporal evolution of metabolic and inflammatory parameters would be valuable to understand their effect on each other. In this study, we evaluated 14 inflammatory markers, as well as metabolic and body composition parameters in subjects with obesity at baseline, 3 months, and 6 months after BMS. We describe the timing of both the systemic metabolic and inflammatory changes and the impact of metabolic alterations on the resolution of systemic inflammation. As well, we integrated a detailed description of the molecular mechanisms of in-vitro and animal model studies to explain our findings, which are contained in an original figure.

## Results

The demographic characteristics of the cohort are summarized in Table 1. Thirty-two patients undergoing BMS in our center were recruited and followed up in appointments at 3 months and 6 months after surgery.

**Table 1:** Demographic description of the cohort of patients.

Variable		
Age (years)	38.9 (± 10.0)	38.9 (± 10.0)
Gender	Female	24 (75%)
	Male	8 (25%)
Surgical procedure	RYGB	20 (62.5%)
	SG	12 (37.5%)

Data presented as mean (standard deviation) or as *n* (percentage).

Most of the patients in the cohort were female (75%). RYGB was more frequent than SG as surgical procedure (62.5% vs 37.5%). Data regarding the specific changes in biochemical, inflammatory and anthropometric variables depending on the type of surgery performed can be consulted in the supplementary material. However, as both patients that underwent RYGB or SG showed similar trends in the magnitude of weight loss and in most of the other evaluated parameters, we present this analysis as a one whole group.

**Figure 1:**
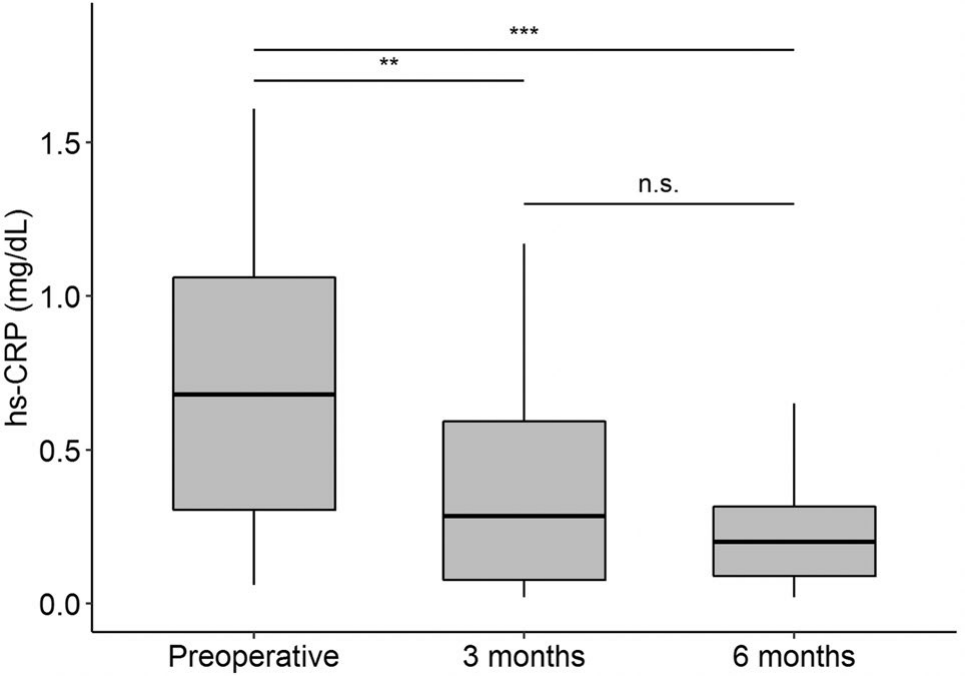
Changes in the levels of hs-CRP before and after bariatric surgery. n = 32; hs-CRP: high sensitivity C-reactive protein; **p-value < 0.01, ***p-value < 0.001, n.s.: non-significant; Friedman test with Nemenyi post-hoc pairwise comparison test. This figure was made with the help of Tidyverse and Hmisc packages in R software.

Table 2 shows the changes in anthropometric, body composition, and biochemical parameters observed through the three time-points. Significant weight loss was observed 3 months after BMS (*p*-value < 0.001), with a further decrease from 3 to 6 months (*p*-value < 0.001). BMI, fat mass, and body fat percentage followed a similar trend. A small significant reduction in fat-free mass was found 3 months after the surgery (*p*-value < 0.001) with no further variations at 6 months. Significant changes in the metabolic parameters were shown 3 months after BMS compared with baseline levels. A statistically significant reduction in the triglyceride (*p*-value = 0.003), total cholesterol (*p*-value = 0.001), high-density lipoprotein cholesterol (HDL-c), and fasting insulin levels (*p*-value < 0.001), as well as the Homeostatic Model Assessment for Insulin Resistance (HOMA-IR) index (*p*-value < 0.001) was obtained 3 months after the surgery, with no additional significant modifications. Low density lipoprotein cholesterol (LDL-c) levels decreased significantly 6 months after the surgery compared with baseline values (*p*-value = 0.002). Although the concentration of apolipoprotein A declined 3 months after the surgery (*p*-value < 0.001), its levels increased later during the time-period between 3 to 6 months (*p*-value = 0.011). No changes were observed for fasting glucose levels at any time-point (p-value = 0.296). As shown in Fig. 1, a significant decrease in hs-CRP levels was found 3 months after BMS compared with basal levels (*p*-value = 0.009). After a follow-up period of 6 months, 71.9% of the patients reached hs-CRP levels below 0.3 mg/dL, which is the cut-off value for cardiovascular risk established by the American Heart Association (AHA)^18^.

**Table 2:** Anthropometric measurements, body composition, and biochemical parameters of the patients before and after bariatric surgery.

Variable	Preoperative	3-month follow-up	6-month follow-up
BMI (kg/m^2^)	41 (37.7–45.3)	32.6 (29.1–35.8)*	29.5 (26.3–33.3)*^#^
Weight (kg)	108.7 (98.5–120.4)	87.1 (78.3–95.3)*	80 (71–88.4)*^#^
Fat mass (kg)	54.3 (47.4–61.4)	39.4 (30.1–47)*	29.2 (22.8–39.8)*^#^
Fat-free mass (kg)	52.8 (48.9–62.8)	49.5 (44.9–55.9)*	47.1 (44.4–54.2)*
Fat mass (%)	50.2 (48.9–52.9)	43.9 (40.6–46.9)*	37 (34.6–42.3)*^#^
Chol (mg/dL)	195 (169.5–214.8)	165 (143.8–184)*	162 (135.5–184.5)*
TG (mg/dL)	123 (88–170)	94.5 (67.5–120.2)*	84 (70–105.2)*
HDL (mg/dL)	45.6 (38.1–56.7)	40 (36.6–49.7)*	43.6 (38.6–50.5)
LDL (mg/dL)	112.8 (102.3–135.5)	98.2 (82.4–118.8)	100.3 (76–115.6)*
Apo A (mg/dL)	142.5 (125.2–165.8)	124.5 (117.2–147.2)*	134.5 (126.8–147.2)^#^
Apo B (mg/dL)	103.5 (88.5–112)	85 (75.8–94)*	80 (70–94.2)*
Glucose (mg/dL)	82 (78.8–96.5)	81 (74–84)	79 (76.2–84)
Insulin (mU/L)	15.9 (10.7–20.2)	6.1 (4.9–8.3)*	5.4 (4.7–7.4)*
HOMA-IR	3.4 (1.8–4.5)	1.3 (0.9–1.8)*	1.1 (1–1.5)*
hs-CRP (mg/dL)	0.7 (0.3–1.1)	0.3 (0.1–0.6)*	0.2 (0.1–0.3)*

*n* = 32. Data summarized as median (IQR), Apo: Apolipoprotein, BMI: Body Mass Index, HDL-c: High Density Lipoprotein Cholesterol, HOMA-IR: Homeostatic Model Assessment for Insulin Resistance, IQR: Interquartile Range, LDL-c: Low Density Lipoprotein Cholesterol, hs-CRP: high sensitivity C-reactive protein,*p-value < 0.05 vs Preoperative, ^#^*p*-value < 0.05 vs 3-month follow-up; Friedman test with post-hoc pairwise Nemenyi test.

The plasma concentration of cytokines before and after BMS are reported in Table 3. Concentrations of the classic proinflammatory cytokines IL-1β, tumor necrosis factor alpha (TNF-α), IL-6, and IL-8 decreased significantly until 6 months after the surgery (*p*-value < 0.001). Monocyte chemotactic factor (MCP-1) presented a trend towards decreased levels at 6 months that did not reach statistical significance. IL-10 levels declined significantly at the 3-month follow-up measurement (*p*-value = 0.028), further decreasing 6 months after surgery (*p*-value = 0.046). The concentrations of T-helper lymphocyte type 1 (Th1) signature cytokine interferon gamma (IFN-γ) and of Th1-promoter cytokine IL-12 were significantly reduced 6 months after the procedure(*p*-value = 0.001; 0.008, respectively) compared with preoperative values. The concentration of IL-18, another Th1 promoter, declined at 3-months follow-up (*p*-value = 0.001), decreasing further at 6-months in comparison to preoperative (*p*-value < 0.001) and 3-months’ value (*p*-value = 0.016). Although a slight decrease in the levels of T-helper lymphocyte type 17 (Th17) signature cytokine IL-17 was observed, it did not reach statistical significance, while Th17-promoter IL-23 was significantly reduced 6 months after BMS (*p*-value < 0.001). Even though a direct measure of T-helper lymphocyte type 2 (Th2) signature cytokines was not evaluated, there were no significant changes in Th2-promoter IL-33. The concentration of interferon apha-2 (IFN-α2) declined significantly 6 months after the procedure (*p*-value < 0.001).

**Table 3:** Plasma cytokine concentrations in the patients before and after bariatric surgery.

Cytokine (pg/mL)	Preoperative	3-month follow-up	6-month follow-up
IL-1β	2.4 (0.3–2.9)	1.1 (0.3–2.4)	0.3 (0.1–2.4)*
IFN-α2	2.3 (0.5–2.6)	2.1 (0.3–2.7)	1.1 (0.3–2.4)*^#^
IFN-γ	2.4 (1.2–3.3)	1.2 (0.7–2.4)	1 (0.5–2.4)*
TNF-α	3.9 (1.6–5.9)	3 (1.6–4.8)	1.7 (0.8–3.3)*^#^
MCP-1	197.2 (144.4–252.8)	185.6 (151.1–231.1)	181.8 (142.9–243.8)
IL-6	4.1 (2.2–6.8)	3 (1.9–4.9)	2.4 (0.9–3.7)*^#^
IL-8	4 (1.5–5.8)	3.2 (1.9–4.6)	2 (0.6–2.5)*^#^
IL-10	1.8 (1.1–2.7)	1.4 (0.7–2.4)*	0.7 (0.4–2.4)*^#^
IL-12	0.7 (0.3–1.4)	0.5 (0.3–1.2)	0.3 (0.3–1)*^#^
IL-17	3.7 (1.9–8.2)	2.3 (1.8–5.4)	2.5 (1.9–3.7)
IL-18	221.7 (134–278.2)	122.1 (98.4–193)*	104.8 (52.4–155.6)*^#^
IL-23	10.1 (3.2–12.6)	5.7 (2.1–11.3)	2.1 (1.9–5.8)*^#^
IL-33	0.3 (0.2–0.3)	0.3 (0.3–0.5)	0.3 (0.2–0.3)

*n* = 32. IFN: Interferon, IL: Interleukin, MCP: Monocyte Chemoattractant Protein, TNF: Tumor Necrosis Factor. Data is presented as median (IQR), *p-value < 0.05 vs preoperative, ^#^p-value < 0.05 vs 3-month follow-up. Friedman test with post-hoc pairwise Nemenyi test.

**Figure 2:**
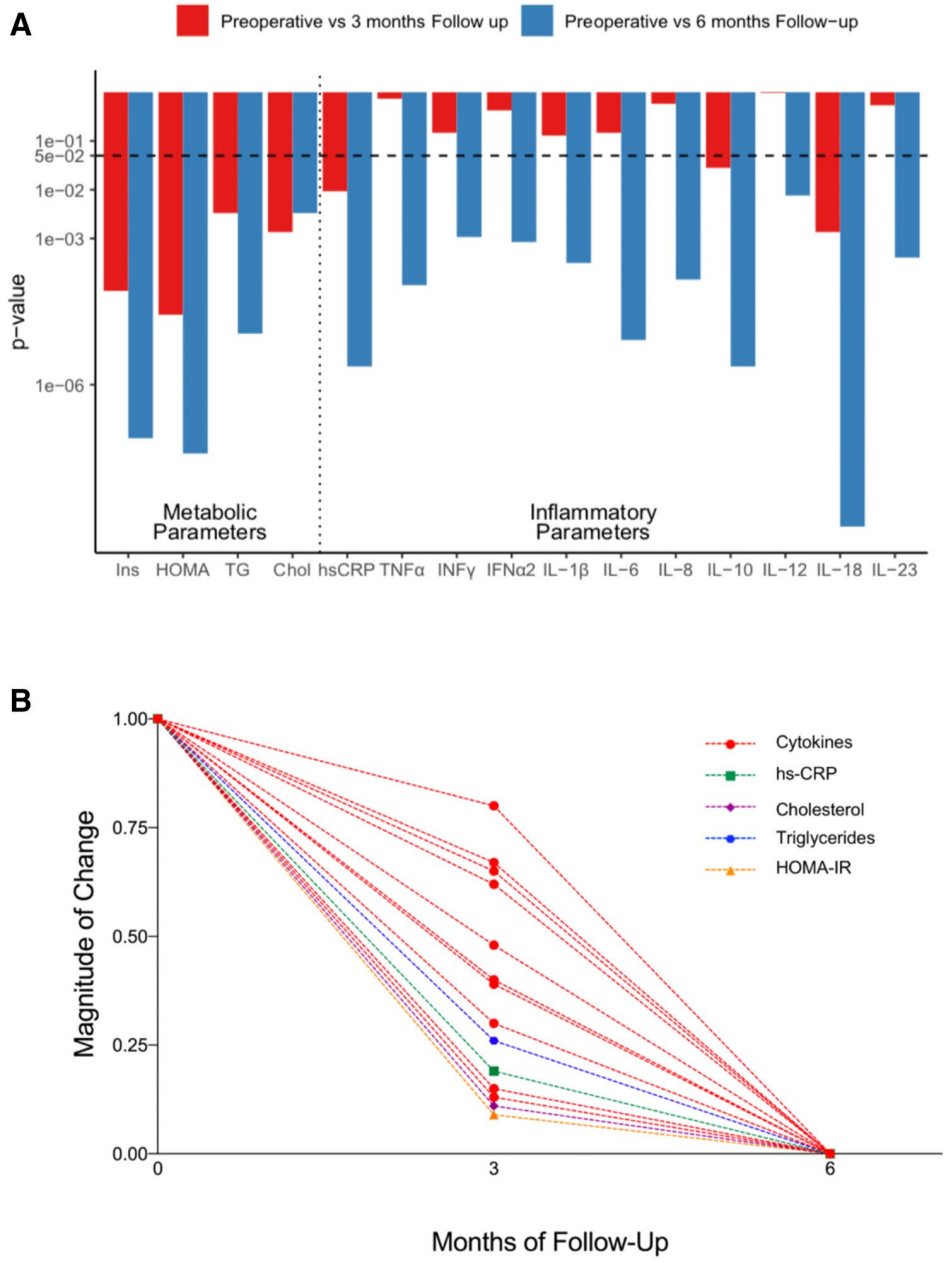
Metabolic normalization precedes resolution of systemic inflammation. (**A**) Comparison of the p-values of the metabolic and inflammatory markers at 3 and 6 months post-surgery versus preoperatory values. The dashed line represents the cut-off for statistical significance (p-value = 0.05). (**B**) Median values at each time point after normalization, represented as the magnitude of change in each variable. IFN: Interferon, IL: Interleukin, TNF: Tumor Necrosis Factor. HOMA-IR: Homeostatic Model Assessment for Insulin Resistance, Chol: Total cholesterol, hs-CRP: high sensitivity C-reactive protein. This figure was made with the help of Tidyverse and Hmisc packages in R software.

Analysis of Tables 2 and 3 shows that while most of the metabolic improvement regarding insulin resistance and the lipid profile occurred during the first three months after BMS, the changes in inflammatory markers showed a non-significant fall at this time point. The significant decline in the circulating levels of inflammatory markers took place until 6 months after the surgery. This is graphically represented in Fig. 2.

**Figure 3:**
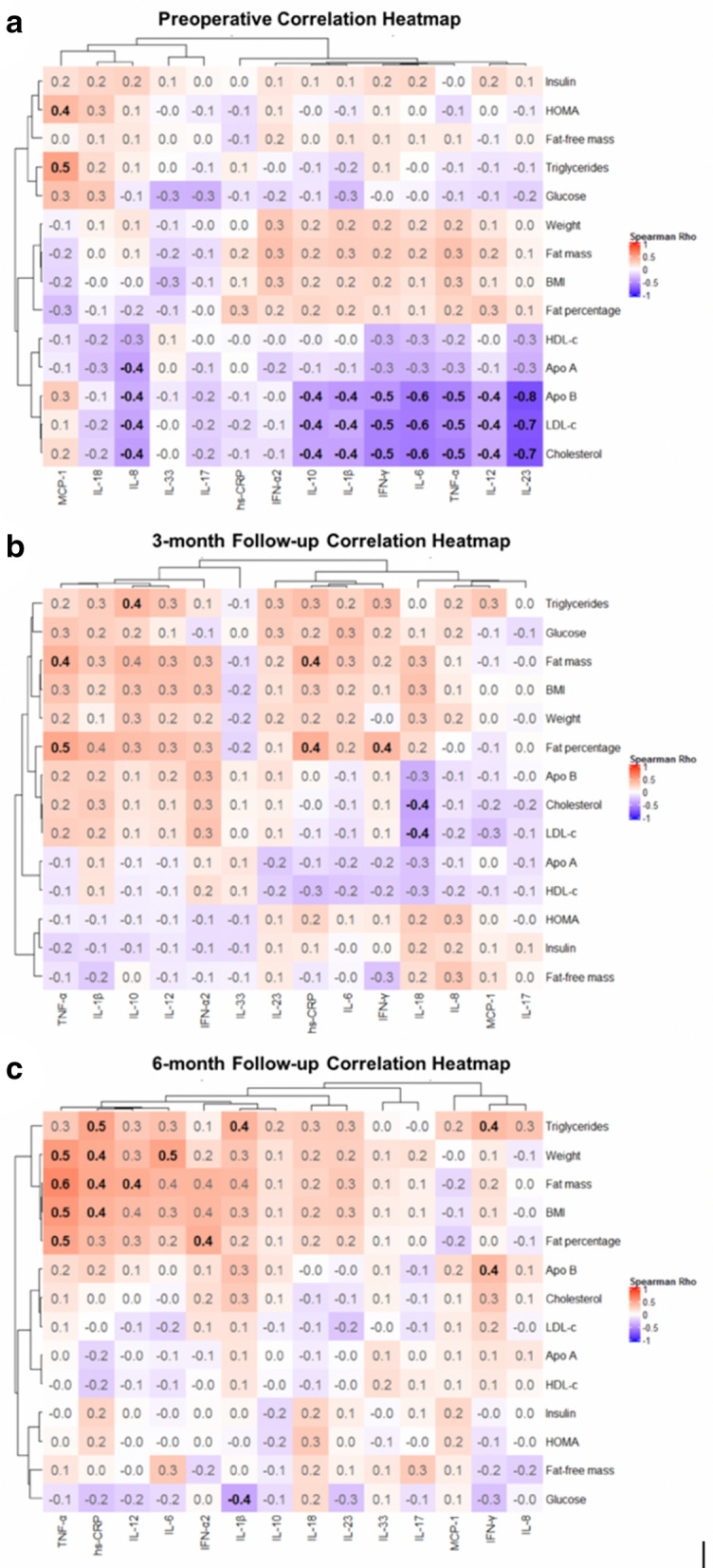
Correlation heatmaps: Inflammatory markers, anthropometric indicators, body composition parameters, and biochemical variables. n = 32. Spearman’s rho rank correlation coefficient is rounded to one decimal and shown in black and bold font for statistically significant findings (*p*-value < 0.05). Red squares represent positive values; Blue squares represent negative values. (**a**) Preoperative; (**b**) Postoperative 3-month follow-up; (**c**) Postoperative 6-month follow-up. Apo: Apolipoprotein, BMI: Body Mass Index, HDL-c: High Density Lipoprotein Cholesterol, HOMA-IR: Homeostatic Model Assessment for Insulin Resistance, LDL-c: Low Density Lipoprotein Cholesterol, hs-CRP: high sensitivity C-reactive protein, IFN: Interferon, IL: Interleukin, MCP: Monocyte Chemoattractant Protein, TNF: Tumor Necrosis Factor. This figure was made with the help of Complex Heatmaps Package in R software.

Figure 3 shows the bivariate correlation analysis performed separately for each time-point: preoperatively, 3-month, and 6-month follow-ups. In the preoperative period (Fig. 3A), IL-1β, IFN-γ, IL-6, TNF-α, IL-12, IL-23, and IL-10 exhibited a statistically significant inverse correlation with total cholesterol (*p* < 0.001–*p* = 0.037), LDL-c (*p* < 0.001–*p* = 0.042), and apolipoprotein B (Apo B) concentrations (*p* < 0.001–*p* = 0.041). IL-8 was also inversely associated with total cholesterol (*p* = 0.017), LDL-c (*p*- = 0.029), Apo B (*p* = 0.048), and apolipoprotein A (Apo A) (*p* = 0.028). Neither hs-CRP nor IFN-α2 concentrations were associated with cholesterol levels. On the other hand, MCP-1 was positively correlated with both the HOMA-IR index (*p*-value = 0.043) and triglyceride levels (*p* = 0.006). No significant association was found between anthropometric variables and cytokines circulating concentrations.

At the 3-month follow-up period, some significant associations were found (Fig. 3B). IL-18 was inversely associated with total cholesterol (*p* = 0.026) and LDL-c levels (*p* = 0.035). Fat mass correlated directly with both hs-CRP (*p*- = 0.049) and TNF-α concentrations (*p* = 0.033), while fat percentage was associated with hs-CRP (*p* = 0.027), TNF-α (*p*- = 0.014), and IFN-γ levels (*p*-value = 0.049). IL-10 significantly correlated with serum triglycerides levels (*p* = 0.041). In addition, TNF-α, IL-1β, IL-10, IL-12, IFN-α2, hs-CRP, IL-6, and IFN-γ showed a trend that did not reach statistical significance toward positive correlations with triglycerides and glucose levels, fat mass, BMI, weight, and body fat percentage.

At the 6-month follow-up period, a significant correlation between hs-CRP and weight, fat mass, BMI, and triglycerides levels (*p* = 0.008–*p* = 0.048) was observed. TNF-α was significantly associated with weight, fat mass, BMI, and body fat percentage (*p* = 0.002–*p* = 0.021). IL-12 significantly correlated with fat mass (*p* = 0.034), and IL-6 significantly correlated with weight (*p* = 0.019). IFN-α2 was positively associated with fat percentage (*p*-value = 0.042), and IFN-γ correlated with triglycerides (*p*-value = 0.047) and Apo B (*p*-value = 0.046) levels. IL-1β showed a positive correlation with triglyceride levels (*p*-value = 0.027).

## Discussion

Early impact of bariatric surgery on metabolic parameters leads to anti-inflammatory outcomes. While both metabolic improvement and systemic inflammation reduction after BMS are well documented, the temporal relationship between them has received relatively less attention. We noticed that in our cohort, metabolic parameters such as insulin, HOMA-IR, and triglycerides were predominantly reduced at 3 months, with relatively stable levels from 3 to 6 months after the surgery. In contrast, for most inflammatory mediators, a significant reduction was observed until 6 months after the procedure, with the notable exceptions of hs-CRP and IL-18, that were mainly decreased during the first three postoperative months. This pattern suggests that metabolic improvement could be a determining factor for inflammation resolution to occur. In terms of temporality, other studies have also found similar results, with reductions in most inflammatory markers predominantly after 6 months of bariatric surgery. The exceptions were reactive oxygen species (ROS), MCP-1, and hs-CRP that presented early reductions at 3 months or less after the procedure^19–21^. Accordingly, several studies have shown absence of significant alterations of inflammatory markers or poor relationships with metabolic modifications in early stages after BMS^20,22^. In this sense, metabolic improvement seems to be an early change after bariatric surgery that may favor obesity-induced inflammation resolution. This has been evidenced in a study conducted in patients with obesity polycystic ovary syndrome (PCOS), an entity characterized by the presence of multiple metabolic disturbances including insulin resistance, which reported that improvement in inflammatory markers such as hs-CRP was slower in patients with PCOS undergoing bariatric surgery in comparison with non-PCOS counterparts^23^. In addition, Yadav et al. described a study focused on the effect of time in the evolution of metabolic and inflammatory parameters after RYGB in 37 patients with obesity (17 diabetic vs 20 non-diabetic). The authors concluded that time after surgery had a strong effect on the reduction of both HOMA-IR and hs-CRP circulating levels during the first 6 months, with no further change from 6 to 12 months. For TNF-α, the effect of time was significant only from 6 to 12 months after the surgery^24^, indicating TNF-α resolution is a relatively late event after BMS, as seen in our study. Concerning TNF- α, a meta-analysis indicated that at least 12 months are required before a consistent decrease in its levels was observed^14^. While we found a reduction in TNF-α and other inflammatory mediators, this timeline is consistent with our findings regarding that improvement in insulin resistance precedes inflammation resolution.

Despite the differences in time-points assessed by diverse studies, there is an overall consensus that insulin resistance reduction and other metabolic benefits can take place as early as 1 week after RYGB, even when a notable reduction in weight is not yet observed^25^. Consistently, our results showed a significant improvement in most of the metabolic and anthropometric parameters as early as the 3-month follow up. In agreement with our results, previous studies have reported marked increases in insulin sensitivity and glucose oral tolerance, reductions in fasting plasma glucose, visceral adipose tissue, and glucose concentrations as early effects of BMS, independently of weight-loss^26,27^. Consistently, we observed a significant reduction on both BMI and fat mass after 3 months of BMS that were sustained even after 6 months of follow-up. Although the implicated mechanisms in this early metabolic response are not completely understood, the modulation of gastrointestinal hormones and adipokines such as GLP-1, peptide YY, cholecystokinin, oxyntomodulin, and leptin, implicated in the energy balance, glucose metabolism, and satiety have been associated to short-term metabolic outcomes of bariatric surgery^28^. As suggested by Sams et al., although there are many proposed mechanisms about the early metabolic impact of BMS, the resolution of metabolic disturbances implicate an early phase of improvement in insulin sensitivity, followed by a late phase of a decreased inflammatory response^29^.

The hypothesis that early metabolic changes after BMS can reduce pro-inflammatory markers might be supported by evidence from preclinical studies reporting that in obesity, disturbances in lipoprotein metabolism and glucose homeostasis have shown to drive pro-inflammatory activation and differentiation of macrophages and other immune cells. These responses have been described to occur through multiple mechanisms, including the production of oxidative stress, alterations in cellular redox potential, and overexpression of key regulators of inflammation such as nuclear factor kB (NF-kB) and Toll like receptors (TLR’s)^30,31^. In addition, murine models have associated NF-kB with the induction of pro-inflammatory cytokines such as TNF-α and IL-6 and with increased energy expenditure^32,33^. As well, TNF-α and IL-6 knockout mouse models have shown positive energy balance^34,35^, suggesting that energy accumulation, observed in obesity, induces chronic inflammation, which in turn, promotes energy expenditure as a compensatory mechanism^36^. Therefore, initial positive metabolic outcomes on energy balance, glucose homeostasis, satiety, calorie intake, lipid metabolism, and insulin sensitivity after BMS may be needed to obtain later improvements in inflammatory parameters. Triglycerides and other lipid species have been described to have pro-inflammatory properties by facilitating the activation of NFkB and c-jun N-temrinal protein kinase 1 (JNK1) signaling pathways, which stimulate the release of pro-inflammatory cytokines such as TNF-α in various cell lineages, namely macrophages, adipocytes, and hepatocytes^31^. As well, lipotoxicity induced by obesity has been associated with the production of ROS, which can lead to the activation of NLR family pyrin domain containing 3 (NLRP3), a potent stimulator of the inflammasome signaling whose primary effect is the production of IL-1β^37^. Therefore, improvements of lipid profiles in patients with obesity after undergoing BMS may play a key role in the reduction of inflammatory markers. Consistently, our results showed that fat mass, body fat percentage, and triglyceride levels presented positive correlations with hs-CPR, TNF-α, and IFN-γ concentrations, suggesting that the lower fat mass content induced a decrease in proinflammatory cytokines. Later on, 6 months after bariatric surgery, circulating levels of TNF-α, hs-CRP, IL-12, IL-6, and IFN-α2 showed strong positive correlations with triglyceride levels, weight, fat mass, BMI, and fat percentage. Additionally, IFN-γ concentration showed a positive association with triglyceride and Apo B levels. We also found a significant correlation between serum triglyceride levels and IL-1β concentration at 6 months after BMS, suggesting that the reduction in triglyceride levels, may contribute to the reduction in IL-1β and other pro-inflammatory cytokine levels. These results show that patients that achieved the lowest body fat mass and triglyceride levels after surgery, also presented lower levels of inflammation. In these context, certain key metabolic regulators such as PPAR-α, whose expression in the liver was shown to increase after RYGB and has been implicated in the downregulation of TNF-α mRNA and hepatic inflammatory infiltrates in preclinical models^38,39^, could be a potential link between lipid disturbance and inflammation resolution after BMS.

Total cholesterol, LDL-c and Apo B have usually been associated with inflammation^40^. However, in the preoperative state, our results showed that total cholesterol, LDL-c, and Apo B were negatively associated with IL-1β, IFN-γ, IL-6, TNF-α, IL-12, IL-23, and IL-10. These findings could be explained by an anti-inflammatory effect of cholesterol, as reported by Spann N.J., et al. Knocked-out mice for LDL receptor presented increased cholesterol accumulation in peritoneal macrophages, which downregulated the expression of inflammatory response genes, such as il1b, through inhibition of cholesterol synthesis pathways^41^. This study suggests that hypercholesterolemia, in some circumstances, might induce an anti-inflammatory effect through the suppression of its biosynthetic pathways. Interestingly, these correlations were not maintained after BMS when significant improvements in these markers occurred.

It is well known that abnormalities in glucose homeostasis can lead to chronic low-grade inflammation. Potential explanations for this mechanism include higher production of ROS and activation of TLR2, TLR4, and receptors for advanced glycation products (RAGES), which in turn mediate the expression of NF-kB canonical signaling involved in the upregulation of pro-inflammatory products^42^. Thus, previous studies have suggested that insulin resistance resolution could be a factor driving the reduction in systemic inflammation by showing significant reductions in HOMA-IR before considerable reductions in the levels of IL-6, MCP-1, IL-18, TNF-α^19,43,44^. Consistently, our results showed a similar pattern of evolution. However, associations between HOMA-IR and pro-inflammatory markers remain controversial. For example, significant inverse correlations between preoperative HOMA-IR and CRP levels reduction after BMS have been described^23^. On the other hand, in a study conducted in 36 patients with metabolic syndrome that underwent RYGB, no significant correlations between HOMA-IR and inflammatory markers such as CRP were found before surgery, although a significant decrease in this parameter was found at 6 weeks and 52 weeks follow up^44^. In our study, despite the lack of correlation between HOMA-IR and the inflammatory markers after BMS, the resolution of insulin resistance occurred well before that of the inflammatory mediators. As stated in a systematic review conducted in 2020, these discrepancies, along published studies, can be explained by the severity of insulin resistance, inherent differences in the study populations, and variability in the quantification techniques of inflammatory mediators^45^.

Possible mechanisms involved in the systemic inflammation associated with obesity, and potential mechanisms involved in late-onset decreased inflammatory markers after bariatric surgery. As depicted in the previous discussion, early metabolic changes after BMS are capable of triggering potential anti-inflammatory mechanisms and also of stimulating weight loss, which in turn, can inhibit the expression of pro-inflammatory molecules in later stages. In this regard, several pathophysiological mechanisms have been proposed to explain the systemic inflammation associated with adiposity. For example, a study in subjects with obesity demonstrated an association of adipocyte size and apoptosis with crown-like structures (CLS) around necrotic adipocytes^46^. Furthermore, in a pooled analysis, the adipose tissue of mice with obesity presented higher expression of TNF-α compared with their lean controls, and the adipocyte cross-sectional area correlated with macrophage content in both visceral and subcutaneous adipose tissue^47^. Mice with high fat diet (HFD)-induced obesity have also been reported to present an increased number of macrophages, CD4+, and CD8+ T cells in adipose tissue, along with augmented expression of IFN-γ mRNA and IFN-γ secretion when compared with lean mice^48^. Our results showed higher plasma levels of IL-6, IL-1β, TNF-α, IL-8, IL-12, and IL-18 in subjects with obesity before bariatric surgery when compared with the postoperative state. In this context, although we did not evaluate directly the number of these cells, our results suggest that when fat mass was at its peak during the preoperative state, our cohort of patients with obesity might have presented CLS agglomerates. CLS agglomerates, in turn, might play a significant role not only in the secretion of IFN-γ and TNF-α as a result of Th1 cell activity, but also in the obesity-associated systemic inflammation present before the surgery. After BMS-induced weight loss, adipose tissue infiltration by macrophages and CLS density have been shown to be reduced after 3 months of the procedure in 17 subjects with morbid obesity^20^. In another study, the adipocyte area was significantly reduced in both diabetic patients with obesity and matched non-diabetics, and CLS density in subcutaneous adipose tissue (ScAT) was significantly reduced in the diabetic group 1 year after bariatric surgery^49^. These reports indicate that reduction in adipocyte hypertrophy after bariatric surgery, which results in decreased CLS formation, may be an important mechanism for the decrement in systemic inflammation after bariatric surgery-induced weight loss, as observed in our population.

Although, this mechanism may explain in part the reduction in pro-inflammatory cytokines, several trends and differences have been found across previously published studies. Our results showed a significant reduction in IL-6, IL-8, and TNF-α concentration 6 months after the surgery compared with baseline levels. In contrast, a study in 39 patients with obesity reported no significant changes in IL-6 or IL-8 serum concentrations 6 months after bariatric surgery, ascribable to a compensatory effect of physical activity^50^. However, in accordance with our results, in 22 patients with obesity and impaired glucose homeostasis, CRP and IL-6 levels decreased 1 month and 6 months after SG^13^. In addition, a recent meta-analysis that included up to 116 studies also concluded that BMS can reduce TNF-α, CRP, and IL-6 serum concentrations, particularly 1 year after the procedure^14^. Regarding IL-1β, which in our results was also found to be reduced 6 months after the surgery, in a study in which follow-up was carried out only at 12 months after bariatric surgery, 32 subjects with obesity that underwent either RYGB or SG presented lower levels of IL-1β^15^. In contrast, in a study measuring a wide panel of cytokines in humans, no significant differences in IL-1β, IL-8, IL-12, IL-2, IL-4, IL-5, IL-10, and IL-13 concentrations were found 3 months after RYGB in 15 women with obesity, which was attributed to the invasive nature of the surgery^51^. Reduced levels of IL-1β in our population may be explained by results from HFD-induced obesity rat models that underwent RYGB and showed that lower levels on the aforementioned cytokine resulted from diminished inflammasome activation in visceral adipose tissue (VAT)^52^. As discussed previously, this mechanism can be exerted by improvement of insulin sensitivity and lipotoxicity.

Another mechanism possibly involved in the resolution of inflammation is the capacity of BMS to revert microbiota alterations that have been widely described to contribute to augmented pro-inflammatory markers such as TNF-α and IL-6 in obesity^53^. Previous studies have shown that BMS can lead to an increase in microbial gene richness and to reduce intestinal permeability to LPS and therefore, to lower systemic concentrations of TNF-α and IL-1β^54^. As we found a decrease in both markers after BMS, these mechanisms may also contribute together with the metabolic milieu to the explanation of this phenomenon.

Possible effects of bariatric surgery on Th1, Th2 and Th17 cell activity. Data regarding the systemic effect of bariatric surgery on other immune responses, such as the Th1, Th2, and Th17 cell activity and IFN-γ, IL-18, and IL-12 secretion, is scarce. Our results showed a significant decrease in IL-18 concentration 3 months after the surgery with further decline at 6 months, while IFN-γ and IL-12 levels declined 6 months after the procedure compared with preoperative values. IFN-γ, the most important mediator of Th1 response, has been reported to increase in subjects with morbid obesity and to decrease after bariatric surgery. For example, in 97 patients with obesity underwent either RYGB or biliopancreatic diversion with duodenal switch, decreased levels of IFN-γ, hs-CRP, TNF-α, IL-13, IL-6, IL-1ra, C3, and leptin were described compared with baseline levels at 1-year follow-up^55^. The authors proposed that the reduced inflammation after bariatric surgery might be attributed both to the reduction in adipose tissue mass and to decreased LPS translocation^55^. These findings suggest that Th1 cells, which induce the secretion of IFN-γ, might play a role in the inflammation present in our patients. After BMS, fat mass reduction and decreased intestinal permeability with subsequent lower LPS plasma levels, might be responsible for the significant decline in IFN-γ concentration. Concerning IL-18, an inducing Th1 pro-inflammatory cytokine, in a study in mice, IL-18 mRNA expression was reduced in VAT after BMS^52^. Our results showed that IL-18 decreased significantly 3 months after the procedure, with further significant decline at 6 months. At the same time, the decrease in both fat mass in kg and body fat percentage peaked at 3 months, with additional reduction 6 months after the bariatric surgery. Considering the previous reports, the lower concentration of IL-18 might be time-dependent and result from decreased fat mass and reduced mRNA expression in VAT. Likewise, increased levels of systemic IL-12, an inductor of Th1 differentiation, were also described in patients with obesity compared with normal-weight subjects^56^, and our results showed a significant decrease in IL-12 levels after bariatric surgery, alongside decreased insulin levels and HOMA-IR index. Considering the results from these studies and given that both IL-18 and IL-12 promote Th1 differentiation, BMS-induced weight loss and improvement in insulin sensitivity in our patients, may favor reduced Th1 cell activity and thus, reduction of these inflammatory markers.

On this point, insulin resistance and obesity have shown to present imbalances in Treg and Th17 activity, as reported in a study that demonstrated lower ratios of Treg/Th17 cells in insulin-resistant rat models compared with healthy controls^57^. Secretion of IL-17, the most important cytokine of Th17 activity, was found diminished three months after RYGB was performed in 9 subjects with obesity with insulin resistance^58^. Although the serum level of IL-23, another product of Th17 cells, in the context of bariatric surgery has not been previously reported, a study conducted in a murine model of diabetes showed that jejunal expression of IL-23 and IL-17 were reduced 4 weeks after performing SG and RYGB^59^. Even though we did not assess the expression of these molecules, our results showed significantly decreased circulating levels of IL-23 and a tendency toward decrease in IL-17. Overall, these results suggest that improvements in insulin resistance and weight loss may favor reduced Th1 and Th17 circulating products.

**Figure 4:**
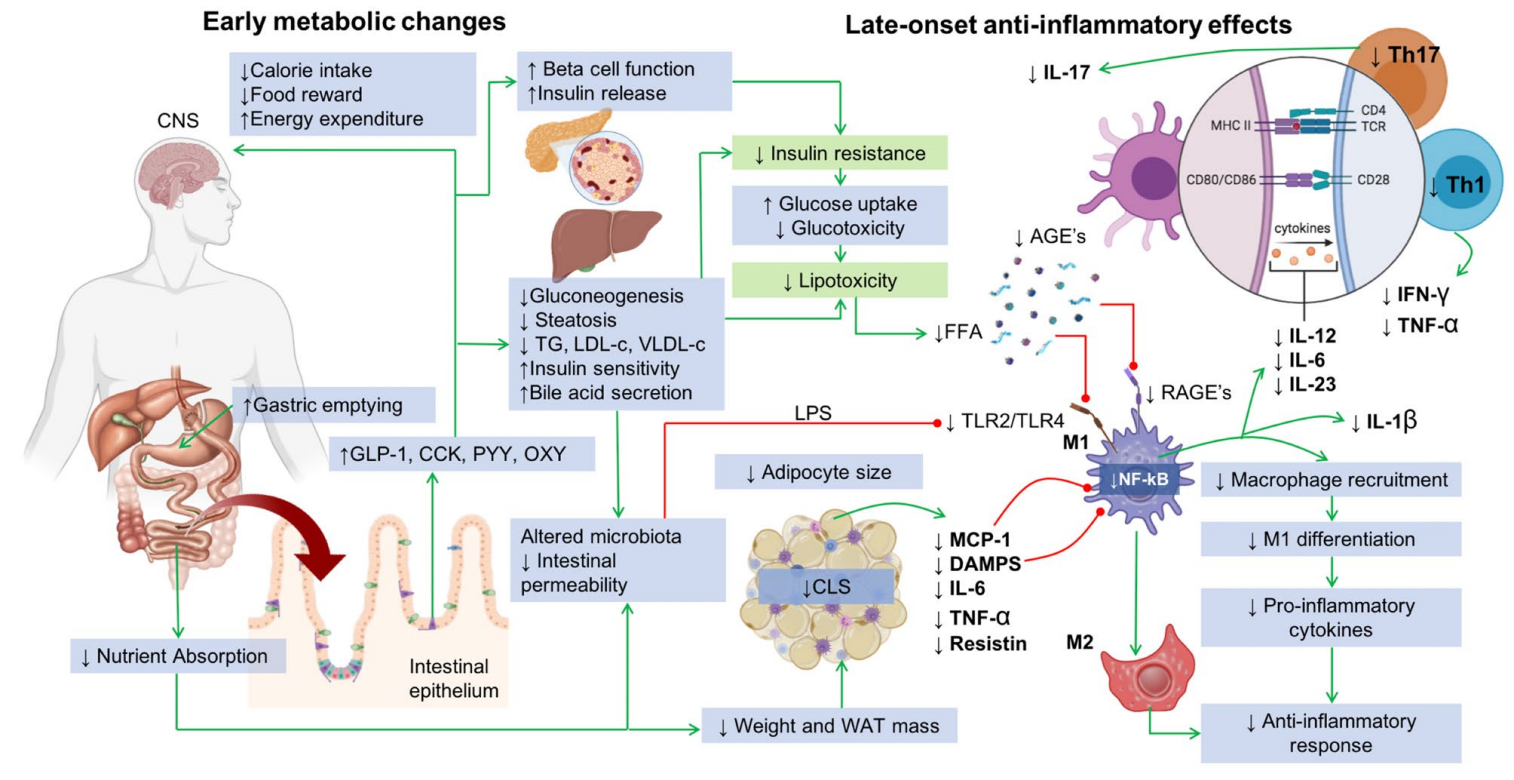
Mechanisms involved in the production of late-onset anti-inflammatory response as a result of early metabolic changes associated with bariatric and metabolic surgery. Green lines indicate pathway activation whereas red lines indicate pathway inhibition. Upper arrows indicate increase and down arrows indicate decrease. GLP-1: Glucagon-like peptide-1; CCK: Cholecystokinin; PYY: Y-Y Peptide; OXY: Oxyntomodulin; TG: Triglycerides; LDL-c: Low density lipoprotein cholesterol; VLDL-c: Very low density lipoprotein cholesterol; LPS: Lipopolysaccharide; FFA: Free fatty acids; TLR: Toll-like receptor; AGE’s: Advanced glycation end products; RAGE’s: Advanced glycation end product receptors; MCP-1: Monocyte chemotactic protein-1; Th1: Type 1 T-helper lymphocyte; Th17: Type 17 T-helper lymphocyte; DAMPS: Damage-associated molecular patterns; M1: M1 Macrophages; M2: M2 Macrophages; IFN-γ: Gamma interferon; TNF-α: Tumor necrosis factor alpha; IL: Interleukin; MHC-II: Mayor histocompatibility complex class II; NF-kB: Nuclear factor kappa-B; TCR: T-Cell receptor; CD: Cluster of differentiation; CLS: Crown-like structures. Figure made by Eder-Luna using Biorender drawing platform.

Overall, we hypothesize that the resolution of systemic inflammation after bariatric surgery-induced weight loss in our patients could be attributed to early metabolic improvements in insulin sensitivity and in the lipoprotein and lipid profile that result as a consequence of initial gastrointestinal functional remodeling and hormonal response. These metabolic changes facilitate future weight loss that further decreases the inflammatory response by reducing fat mass and adipocyte hypertrophy, with subsequent decline in CLS aggregates and decreased mRNA expression of cytokines in VAT, as demonstrated by others. We provide evidence that cytokines that elicit Th1 and Th17 differentiation are reduced after bariatric surgery. Based on preclinical studies, it is possible that a decrease in the number of circulating Th1 cells induced by diminished IL-12 and IL-18 signaling might ensue. Additionally, other mechanisms such decreased gut permeability, changes in bile acid secretion, and increased expression of PPAR-α in the liver might also contribute. Hypothesized mechanisms that lead to anti-inflammatory response as consequence of BMS are summarized in Fig. 4.

In conclusion, weight loss after bariatric surgery led to reduced systemic inflammation and metabolic improvement. The deleterious lipid profile, glucose and insulin circulating levels, and HOMA-IR index declined significantly 3 months after bariatric surgery compared with preoperative levels, while the concentration of proinflammatory cytokines TNF-α, IL-6, IL-8, and IL-1β, decreased until 6 months after the surgery. Plasma levels of hsCRP declined significantly 3 months after the procedure, with further significant decrease at 6 months compared with levels prior to the surgery. Lower levels of plasma IL-10 after bariatric surgery, alongside its close association with IL-1β, suggest that IL-10 might be secreted in obesity as a compensatory anti-inflammatory mechanism. T cell responses could also be modified by weight loss after bariatric surgery. A decline in the circulating levels of both IL-12 and IL-18, which are Th1 cells promoters, and of IFN-γ levels 6 months after the procedure may indirectly point toward reduced Th1 cell activity or count, while decreased IL-23 concentration might potentially represent lower Th17 cell activity. A decline in serum triglycerides levels at 3 months, which correlated with inflammatory markers, might contribute to the lower levels of hs-CRP, IL-1β, and IFN-γ observed at the 6-month follow-up period, possibly suggesting a role of lipid metabolism in inflammation resolution after weight loss. Given that metabolic markers decreased significantly 3 months after bariatric surgery and the reduction in proinflammatory cytokines occurred 6 months after the procedure, the reduction in glucose concentration, insulin levels, and the HOMA-IR index might also influence the decrease in inflammation. We provide new evidence that IL-23 plasma concentration is reduced after bariatric surgery-induced weight loss. Overall, in our study, the reduction in the plasma concentrations of the Th17-driver IL-23, the Th1-induced IFN-γ, and the observed downward trend in IL-17 after bariatric surgery could theoretically suggest a decrease in Th17 cell activity after weight loss. More studies are required to characterize the role of Th17 cells, its drivers and secreted interleukins in inflammation resolution after weight loss-induced bariatric surgery. The role of lipid metabolism regarding Th17 cells in the context of bariatric surgery should be addressed in future studies. Finally, an interesting insight of this study is that in terms of temporality, metabolic improvement seems to precede resolution of systemic low-grade inflammation after BMS. Therefore, it is plausible to suggest that metabolic changes additionally to weight loss are critical driving mechanisms for the resolution of systemic inflammation.

## Methods

### Population

A prospective study was performed in a cohort of 32 patients with obesity who underwent bariatric surgery either RYGB or SG. Inclusion criteria consisted of age older than 18 years, a BMI above 40 kg/m^2^ or a BMI above 35 kg/m^2^ in the presence of comorbidities. Obesity-associated comorbidities were defined as any of the following: T2DM, arterial hypertension, dyslipidemia, sleep apnea, Pickwickian syndrome, non-alcoholic fatty liver disease, pseudotumor cerebri, gastro-esophageal reflux disease, asthma, chronic venous insufficiency, severe urinary incontinence, osteoarthritis, or a severe decrease in quality of life. Patients recruited must be subsequent patients with at least 1 year of management and care in our obesity clinic. Additionally, patients must have tried to lose weight by a non-surgical method for at least 1 year without positive results and receive positive advice to underwent surgery by a multidisciplinary team consisting of a bariatric surgeon, endocrinologist, registered dietitian and psychiatrist. Exclusion criteria consisted of patients with diagnosed pregnancy, patients with prior BMS, patients with recent abdominal, thoracic, pelvic or obstetric surgery within the last 3 months. Patients with diagnosed with a malignancy within 5 years, anemia, chronic kidney disease, secondary hypertension, known history of malabsorptive gastrointestinal disorders or treated with other investigational therapies for obesity within the last 3 months were also excluded from this study.

### Surgical procedures

The patients underwent RYGB or SG, based on a case-by-case decision between the patient and the surgeon, with no input from the research team. All the procedures were performed by laparoscopy.

### Statement

All experiments and procedures conducted in this study were performed in accordance with relevant guidelines and regulations. Both the ethics and the research institutional committees of Tecnologico de Monterrey approved the study protocol (17 CI 19039003). All patients signed an informed consent form.

### Anthropometric and biochemical measurements

Prior to the surgical procedure and during the 3-month and 6-month follow-up appointments, a certified clinical nutritionist obtained the anthropometric measurements and body composition variables, including weight, BMI, fat mass, fat-free mas, and fat percentage using the InBody120 bioimpedance device. During the same previously established time-points, certified and trained nurse personnel obtained venous blood samples via venipuncture of the antecubital vein. Blood samples were processed within 1 h. Serum was isolated by centrifugation at 2665*g* for 10 min at 4 °C and preserved at − 80 °C until used. Plasma was isolated by centrifugation at 400*g* for 30 min at 20 °C and preserved at − 80 °C until used. The lipid profile was measured by nephelometry (Abbot Laboratories, IL, USA) and fasting glucose by the hexokinase/glucose-6-phosphate dehydrogenase method with Glucose 3L82 reagent (Denka Seiken Co. Ltd., Tokyo, Japan), while fasting insulin was obtained by chemiluminescence, using the Architect Insulin Reagent 8K41-27 kit (Abbot Laboratories, IL, USA). HOMA-IR was calculated by a formula as previously described by others^60^. Hs-CRP was measured by quantitative immunoturbidimetric assay with the CRP Vario 6K26-30 and 6K26-41 kits (Abbott Laboratories, Chicago, IL, USA). The cytokine plasma concentration was obtained by flow cytometry using a bead-based multi-assay (Human Inflammatory Panel 1, LegendPlex).

### Statistical analysis

Distribution of quantitative variables was evaluated with Shapiro–Wilk test. Data comparison between time-points was assessed with non-parametric Friedman test and pairwise post-hoc Nemenyi test. Bivariate associations were evaluated by Spearman’s rho correlation test. The analysis was performed with assistance of the R software version 3.6.1 (R Core Team, Vienna, Austria)^61^ and of Tidyverse^62^, Hmisc^63^, and ComplexHeatmap packages^64^ for this software.

## Supplementary Information

Supplementary Information.

## Data Availability

Tables with the complete correlation analysis for this study can be requested to corresponding author Leticia Elizondo-Montemayor (lelizond@tec.mx). Patients signed an informed consent allowing our research team to use their clinical and biochemical data and so cannot be publicly disclosed. Requests for access to these data should be made to the corresponding author Leticia Elizondo-Montemayor (lelizond@tec.mx).
