# Supplementary material for: Metabolic shift precedes the resolution of inflammation in a cohort of patients undergoing bariatric and metabolic surgery
Source: Sci Rep. 2021 Jun 9;11:12127. doi: 10.1038/s41598-021-91393-y (PMC8190106; doi:10.1038/s41598-021-91393-y)
Supplement: Supplementary file 1 — Supplementary Information. [file 41598_2021_91393_MOESM1_ESM.docx]

**Supplementary Material**

**Metabolic shift precedes the resolution of inflammation in a cohort of patients undergoing bariatric and metabolic surgery**

Jose Romeo Villarreal-Calderon^1,2,4^, Ricardo Cuellar-Tamez^1,4^, Elena C. Castillo^1,4^, Eder Luna-Ceron^1,4^, Gerardo García-Rivas^1,3,4*^ and Leticia Elizondo-Montemayor^1,2,4*^

^1^ Tecnologico de Monterrey. Escuela de Medicina y Ciencias de la Salud, Monterrey, 64710, México.

^2^ Tecnologico de Monterrey. Centro de Investigación en Obesidad y Nutrición Clínica, Monterrey, 64710, México.

^3^ Tecnologico de Monterrey, Centro de Investigación Biomédica, Hospital Zambrano Hellion, TecSalud, San Pedro Garza García, 66278, México.

^4^ Tecnologico de Monterrey. Cardiovascular Medicine and Metabolomics Research Group, Hospital Zambrano Hellion, TecSalud, San Pedro Garza García, 66278, México.

*Correspondence should be addressed to Leticia Elizondo-Montemayor, [lelizond@tec.mx](mailto:lelizond@tec.mx); and Gerardo Garcia-Rivas, [gdejesus@tec.mx](mailto:gdejesus@tec.mx)

| Variable | RYGB (n=20) | SG (n=12) | *P value* |
| --- | --- | --- | --- |
| BMI (kg/m2) | 30.5 (27.2-34.7) | 26.1 (25.2-34) | *p=*0.057 |
| Weight (kg) | 84.7 (78.6-92.4) | 77.6 (64.9-90.5) | *p=*0.277 |
| Fat-free mass (kg) | 50.6 (44.9-59.4) | 45.6(30.0-61.8) | *p=*0.381 |
| Fat mass (%) | 38.7 (34.8-44.6) | 35.6 (32.5-38.3) | *p=*0.262 |
| Chol (mg/dL) | 154 (127-178.5) | 173.5 (157-5-192) | *p=*0.071 |
| TG (mg/dL) | 89.5 (77.5-109.7) | 79.5 (57.7-103.7) | *p=*0.172 |
| HDL (mg/dL) | 40.75 (37.3-47.9) | 50.55 (43.4-64.5) | *p=*0.013* |
| LDL (mg/dL) | 84.4 (71.8-113.8) | 104.8 (87.5-114.1) | *p=*0.069 |
| Apo A (mg/dL) | 133 (120-141) | 145 (131.7-163.7) | *p=*0.025* |
| Apo B (mg/dL) | 73 (70-94) | 88.5 (79.2-96.2) | *p=*0.117 |
| Glucose (mg/dL) | 78.5 (72.5-83.5) | 80.5 (74.7-84.7) | *p=*0.609 |
| Insulin (mU/L) | 5.4 (4.2-7.5) | 5.95 (3.5-9.4) | *p=*0.840 |
| HOMA-IR | 1.15 (1-1.4) | 1.2 (0.62-2.0) | *p=*0.824 |
| hs-CRP (mg/dL) | 0.02(0.07-.45) | 0.18 (0.1-0.2) | *p=*0.795 |

**Supplementary Table 1**. Anthropometric measurements, body composition, and biochemical parameters of the patients undergoing either RYGB or SG after 6 months of follow-up. *n* = 32. Data summarized as median (IQR), Apo: Apolipoprotein, BMI: Body Mass Index, HDL-c: High Density Lipoprotein Cholesterol, HOMA-IR: Homeostatic Model Assessment for Insulin Resistance, IQR: Interquartile Range, LDL-c: Low Density Lipoprotein Cholesterol, hs-CRP: high sensitivity C-reactive protein, RGYB: Roux-Y-gastroplasty, SG: Sleeve gastrectomy.


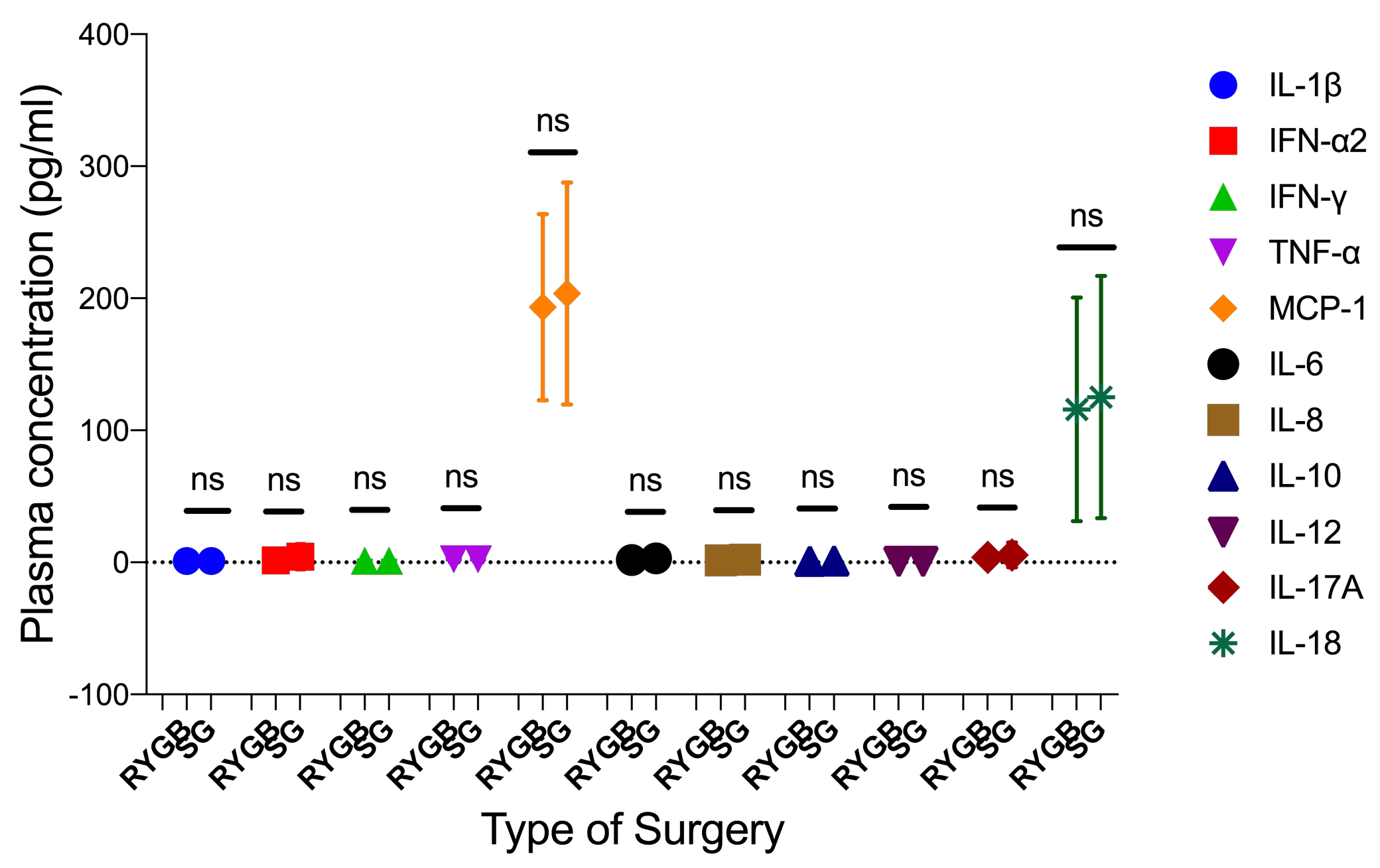


**Supplementary Figure 1**. Cytokine plasma levels of patients undergoing either RYGB or SG after 6 months of follow-up. *n* = 20 and 12 respectively. RYGB: Roux-Y-gastroplasty, SG: Sleeve gastrectomy. IFN: Interferon, IL: Interleukin, MCP: Monocyte Chemoattractant Protein, TNF: Tumor Necrosis Factor. *: p-value < 0.05, ns: non-significant (p>0.05).


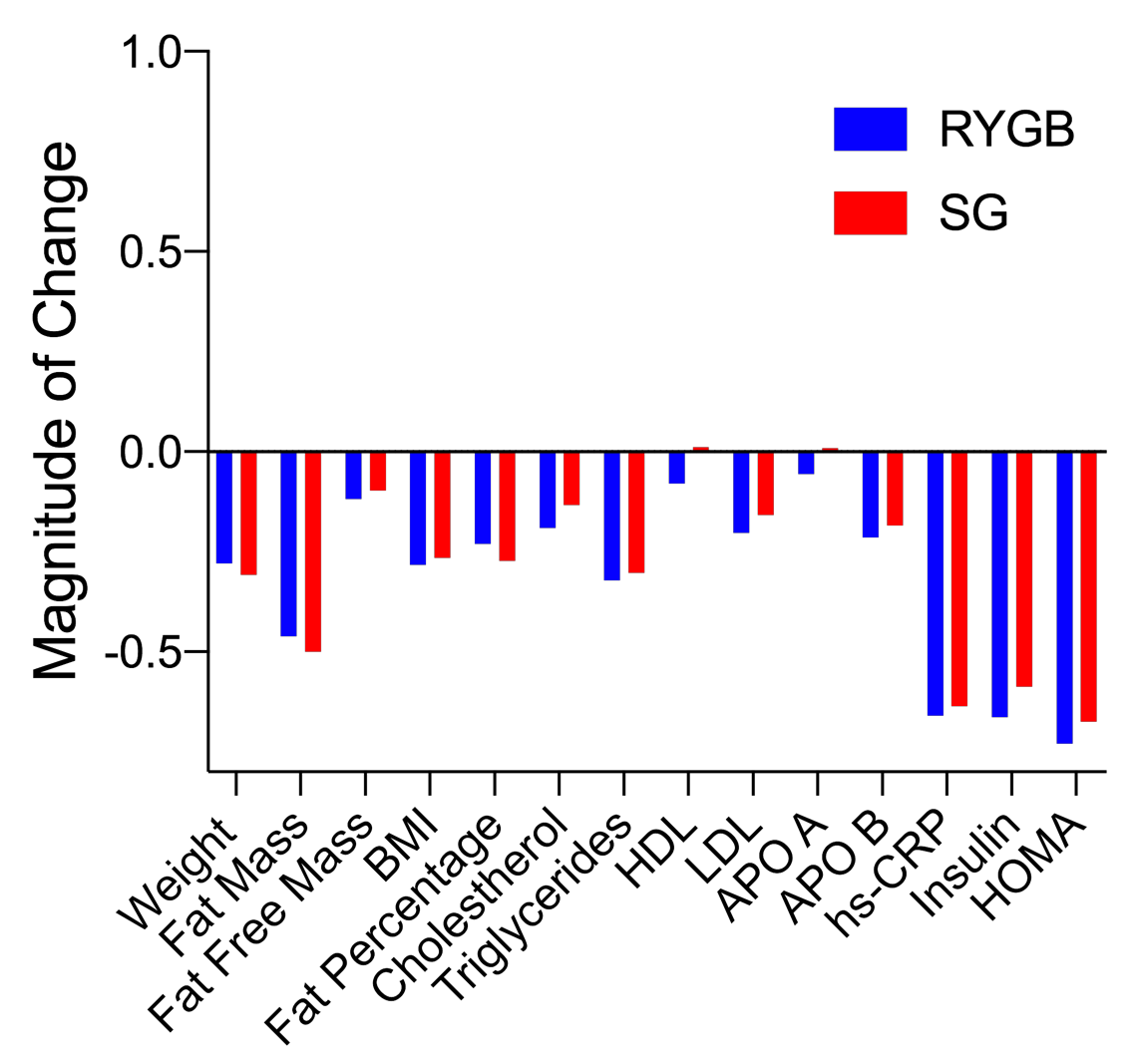


**Supplementary Figure 2**. Magnitude of change of anthropometric and biochemical parameters of patients undergoing either RYGB or SG after 6 months of follow-up. *n* = 20 and 12 respectively. Apo: Apolipoprotein, BMI: Body Mass Index, HDL-c: High Density Lipoprotein Cholesterol, HOMA-IR: Homeostatic Model Assessment for Insulin Resistance, IQR: Interquartile Range, LDL-c: Low Density Lipoprotein Cholesterol, hs-CRP: high sensitivity C-reactive protein, RGYB: Roux-Y-gastroplasty, SG: Sleeve gastrectomy.
